# Perceptional and Socio-Demographic Factors Associated with Household Drinking Water Management Strategies in Rural Puerto Rico

**DOI:** 10.1371/journal.pone.0088059

**Published:** 2014-02-28

**Authors:** Meha Jain, Yili Lim, Javier A. Arce-Nazario, María Uriarte

**Affiliations:** 1 Department of Ecology, Evolution and Environmental Biology, Columbia University, New York, New York, United States of America; 2 Department of Biology, University of Puerto Rico in Cayey, Cayey, Puerto Rico, United States of America; Universitat Rovira i Virgili, Spain

## Abstract

Identifying which factors influence household water management can help policy makers target interventions to improve drinking water quality for communities that may not receive adequate water quality at the tap. We assessed which perceptional and socio-demographic factors are associated with household drinking water management strategies in rural Puerto Rico. Specifically, we examined which factors were associated with household decisions to boil or filter tap water before drinking, or to obtain drinking water from multiple sources. We find that households differ in their management strategies depending on the institution that distributes water (i.e. government PRASA vs community-managed non-PRASA), perceptions of institutional efficacy, and perceptions of water quality. Specifically, households in PRASA communities are more likely to boil and filter their tap water due to perceptions of low water quality. Households in non-PRASA communities are more likely to procure water from multiple sources due to perceptions of institutional inefficacy. Based on informal discussions with community members, we suggest that water quality may be improved if PRASA systems improve the taste and odor of tap water, possibly by allowing for dechlorination prior to distribution, and if non-PRASA systems reduce the turbidity of water at the tap, possibly by increasing the degree of chlorination and filtering prior to distribution. Future studies should examine objective water quality standards to identify whether current management strategies are effective at improving water quality prior to consumption.

## Introduction

Over 700 million people across the globe do not have access to clean drinking water, leading to high levels of chronic waterborne illnesses [1]–[3]. This is particularly problematic in rural communities that do not receive adequately treated water from government facilities and may not have access to appropriate technologies to treat water locally [4], [5]. Scientists and policymakers have long considered the best ways to improve access to potable water, yet identifying the most effective ways to manage drinking water is difficult given that it is typically managed by multiple public and private agencies [6]–[8]. Drinking water is often extracted and treated at different spatial scales (e.g. regional, watershed, and household level), resulting in management by various stakeholders that act at each of these scales (e.g. governmental, private, and household sectors; [9], [10]. Given the complexity of drinking water management, policy makers and agencies (e.g. World Health Organization) over the past decade have increasingly recognized the importance of household water management, particularly in regions where government and community water treatment facilities are ineffective [11], [12]. Households play an important role in determining the water quality experienced by individuals, as households are the last point of management prior to consumption [4], [12].

To target the most successful interventions, it is important to understand the socio-cultural context of current household water management decisions [13]; by understanding how households manage their drinking water and why, policymakers can more effectively target intervention strategies to improve water quality prior to consumption. Though most households in a given community face the same water quality at the tap, some may treat their water prior to consumption while others may not [14], [15]. This variation in household water management is influenced by a variety of factors, including knowledge of water treatment practices prior to distribution, perceptions of water quality at the tap, and socio-demographic characteristics of the decision-maker [14], [16], [17]. For example, previous studies have found that households are more likely to treat their tap water when they believe that government or community treatment facilities are ineffective [18], [19], or when they believe that water quality is low at the tap [15]. While previous studies have examined the importance of these factors individually, few studies have considered these multiple drivers within the same analysis. Doing so is important because it identifies which factors are the most influential for household decision-making. This knowledge can then be used to identify and target interventions that are in line with current household perceptions, which has been shown to result in a greater rate of intervention uptake and success [20].

Our study assesses which factors most strongly influence household water management decisions, specifically whether households filter or boil their tap water prior to consumption or whether they obtain drinking water from multiple sources, in rural Puerto Rico. It is important to understand household water management in this region because previous studies have suggested that broader water management institutions do not always provide adequate water quality at the tap, particularly in rural, mountainous regions that are far from government treatment facilities [21]. There are two broad categories of institutions that manage drinking water for the island's four million people: government-managed Puerto Rico Aqueduct and Sewer Authority (PRASA) systems (which serve approximately 3.8 million people), and private and community non-PRASA systems (which serve approximately 400 communities, or up to 250,000 people), which are found primarily in mountainous regions that are too far to be connected to PRASA treatment facilities [21], [22]. While the non-PRASA category encompasses a range of management strategies, given decentralized management where each community typically develops their own management plan, it is widely believed that non-PRASA communities in general are exposed to low water quality at the tap due to ineffective management of water prior to distribution. The Puerto Rico Department of Health (PRDOH) considers non-PRASA systems to be a health threat since they typically do not comply with federal water quality standards [23]. This is because about fifty percent of non-PRASA systems obtain water from surface sources and there is little or no monitoring of water quality in these communities [24]. Previous studies estimate that 30% of non-PRASA systems lack any water treatment infrastructure [22], and water is not treated consistently even when water infrastructure exists [22], [25]. PRASA systems on the other hand typically filter and chlorinate water at treatment facilities before distribution and provide water quality assessments required by the U.S. Federal Potable Water Standards. Despite centralized management, PRASA systems are often plagued by water shortages and high rates of sediment loading and turbidity, which can result in non-compliances with the US Environmental Protection Agency (EPA) water quality standards [26]. This is because many filtration plants, particularly in mountainous regions, are not equipped to handle water filtration during periods of heavy rainfall [23], which is especially problematic given Puerto Rico's high frequency of tropical storms [27].

Given the possibility of inadequate water treatment by non-PRASA and PRASA facilities, some households have developed management strategies that are thought to improve drinking water quality prior to consumption. These strategies include filtering or boiling tap water or obtaining water from alternate sources like private wells and local markets. In this study, we assessed which perceptional factors that have been postulated to be important in previous literature are most associated with households that undertake water management strategies in rural, mountainous Puerto Rico [15], [18], [19]. Specifically, we predict the following in order of importance:

1. households will have different management techniques depending on whether water is provided by government (PRASA) or community (non-PRASA) institutions likely due to differences in water quality at the tap;
2. households that have problems with institutional water management prior to distribution are more likely to treat water;
3. households are more likely to treat water if they perceive that water from the tap is of low quality;
4. households that have less knowledge about how their water is treated prior to distribution are more likely to treat their water.

We quantify the relative importance of these various factors for household decision-making to better guide future water quality assessments and interventions in rural Puerto Rico. While our results are specific to Puerto Rico, we argue that our methodology can also be implemented in other regions to better understand the drivers of household water management and more effectively target interventions to those households vulnerable to low water quality.

## Methods

### Study site

**Figure 1:**
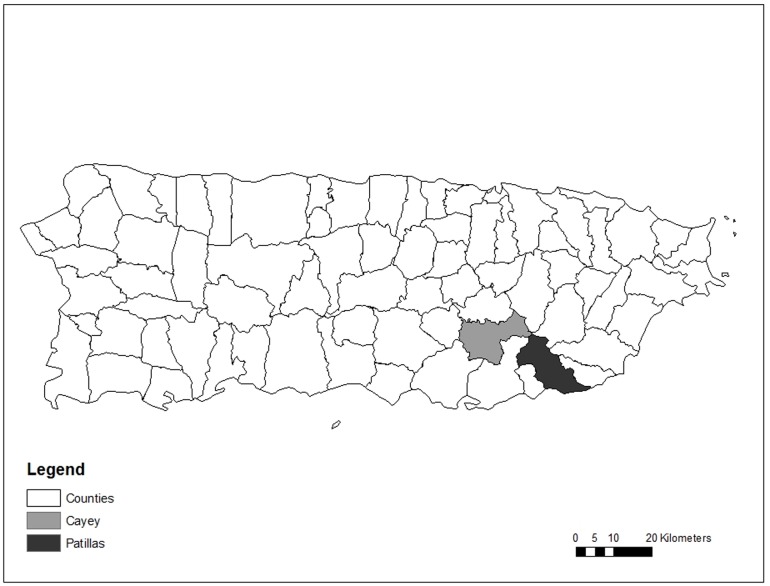
Map of Study Region in Puerto Rico. Municipalities where surveys were conducted are highlighted in gray. We did not list specific communities that we visited to keep the communities we surveyed anonymous.

Data were collected in eight different community sectors within the Cayey Mountain range in Puerto Rico from June to August of 2009. Our study focused on communities in this region because they are thought to be at high risk for low water quality given that they are rural and found in mountainous terrain, which makes them difficult to connect to PRASA treatment facilities. We specifically focused on villages found in Cayey and Patillas municipalities (Figure 1), which contain a large number of non-PRASA communities. Both municipalities are similar in socio-economic and development status. The median household income was $10,923 in Cayey and $9,375 in Patillas in 2000, which were lower than the island average of $13,189 [28]. We selected PRASA and non-PRASA communities that were adjacent to one another in each of the two municipalities. This was possible when we interviewed communities at the boundary where PRASA systems stopped serving communities with piped government water. This paired sampling design reduced possible confounding effects from socio-economic and geographic factors and allowed us to better assess whether households make different decisions based on if PRASA or non-PRASA institutions manage their water. Initial communities (n = 2) were selected based on where our field team had previous experience and knew PRASA and non-PRASA communities were adjacent to one other. We then used a snowball technique and visited additional communities (n = 6) that were suggested to us by the initial community contact [29]. While the communities that we selected for sampling were not entirely selected at random given this snowball technique, we believe that they are representative of the broader region given that each of our four pairs of PRASA and non-PRASA communities were spread across a wide geographic area in the Cayey mountain range (up to 15 km between our four sites).

### Data collection

We surveyed 218 respondents across the eight community sectors considered in our study. Each community sector ranged in size from 50 to 200 households, but to ensure comparability we selected adjacent PRASA and non-PRASA communities that were approximately the same size. We aimed to interview 20 to 30 households in each community, and selected survey households at random distributed equally throughout each community. A summary of the number of survey respondents in PRASA verus non-PRASA communities is given in the supplementary information (Table S1). We then spoke to the household member who answered the door and identified which member of the household was in charge of household water management decisions. If that family member was home, we then conducted the oral structured survey with that family member. If the family member in charge of water management decisions was not at home we skipped that household and did not include it in our survey sample.

#### Ethics statement

Surveys were approved by the Columbia University Institutional Review Board under protocol number IRB-AAAE0079 and informed consent was written. Surveys were conducted in Spanish by local research assistants. We asked all respondents if we could audio record their interviews in order to keep a record of responses and to assist in confirming written responses and only did so if the interviewee gave permission. Our survey instrument contained questions related to whether households undertake any drinking water management prior to consumption, the respondent's perceptions of institutional water management and water quality at the tap, and socio-demographic information for the respondent. Details about each question are listed below, and all data collected were self-reported.

We asked respondents how they managed their drinking water sources prior to consumption, which serves as the dependent variable in our analyses. We grouped responses into two different types of strategies that households may undertake to cope with inadequate water quality. One coping strategy is to increase *the number of drinking water sources* used in the household. Households may diversify sources of drinking water by purchasing bottled water or obtaining drinking water from a personal well. The second coping strategy considered in this study is if households *treat tap water before drinking*. If households believe that their tap water is of inadequate quality, they may filter or boil it before drinking.

We also collected data on the following variables that have been suggested to be important for household water management decisions in previous studies. These variables serve as covariates in our statistical models and we discuss specific data that were collected for each variable of interest. As outlined in the introduction, we believe that management institution type, problems with institutional water management, perceptions of water quality, and knowledge of water treatment will influence household decisions to manage drinking water.

#### Management institution

We considered *the type of institution that manages water* (i.e. *PRASA or non-PRASA*) as a fixed effect because the way that specific institutions manage water may influence household decision-making. This may occur if institutions influence the behavior of households via uniform rules and norms [30]. Institutions may also affect household decision-making if they expose all households in a given community to the same quality of resource. Previous studies have shown that mismanagement of water treatment by institutions may negatively impact water quality experienced by all households within the distribution system [14].

#### Problems with institutional management

As a broad measure of whether households believe that institutions effectively manage water, which has been shown to be important in the previous literature [18], [19], we asked households whether they *have problems with how their water is managed* by PRASA or non-PRASA operators. We predict that respondents who have more problems with institutional management are more likely to treat tap water since they may believe that their water was inadequately treated before distribution.

#### Perceptions of water quality at the tap

Even though all households in a given community are exposed to the same water quality at the tap, varying perceptions may lead to heterogeneous behavior among decision-makers. Previous studies have shown that perceptions of water quality are strong drivers of household water management decisions [15]. To assess water quality perceptions, we asked respondents to rank the *quality of their tap water* on a scale of 1 to 4, where 1 equals poor water quality and 4 equals excellent water quality. We predict that households that believe they have poor water quality are more likely to develop coping strategies.

#### Knowledge of institutional management

Given that previous studies have suggested that increased knowledge of institutional management practices influences individual decision-making [31], we asked respondents whether they *knew how their water was treated* before it is piped to their homes. We predict that households that have less knowledge of how their water was treated by management institutions are more likely to treat water given that they may not trust that their water was treated prior to distribution. Previous studies have suggested a link between increased knowledge, transparency, and trust [32], [33].

#### Socio-economic and demographic variables

Various socio-economic and demographic factors, such as income, age, and gender of the decision-maker, can influence household decisions [34], [35]. We considered the age and gender of the respondent as controls in our analysis, but did not include income in our final models because only half of our interviewees responded to this question. Income data were collected as self-reported annual income for the household in $10,000 US increments (e.g. $10,000–$20,000, $20,000–$30,000, etc.). However, to test whether income may be important for water management decisions in our region, we ran our statistical models on the subset of data with income. We found that the income variable was never significant (p>0.05), suggesting that it is not a significant driver of water management decisions in this region. Furthermore, since we are interested in quantifying the relative importance of various perceptional and socio-demographic factors for decision-making, excluding income from the analysis should not impact our results; instead, it would at most reduce the amount of variance explained by our models.

### Statistical analyses

We conducted three sets of analyses to identify how water management and the drivers of water treatment decisions varied across households in our study. First, we used ANOVA to compare institution types for our two dependent variables of interest: the number of water sources and water treatment. We also compared the distribution of our covariates between institution type using ANOVA analyses. These simple comparisons illustrate whether there were significant differences in coping strategies, perceptions, and socio-demographic factors between households in PRASA and non-PRASA communities.

In a second set of analyses, we used separate logistic regressions to assess the effects of all covariates (Table 1) on the two response variables of interest. To assess whether these covariates have different effects on household decision-making in PRASA and non-PRASA communities, we included interactions between management institution (i.e. PRASA, non-PRASA) and the other covariates. To avoid parameter tradeoffs and clarify interpretation of the results, we dropped covariates that had a correlation >0.4. Based on this criterion, we dropped gender from our analysis. We then conducted stepwise variable selection using AIC_c_ to select the best model [36]. To facilitate the interpretation of effect magnitudes among covariates, all continuous predictors were standardized by subtracting their mean and dividing by twice their standard deviation [37]. Goodness of fit was calculated using the universal goodness of fit le Cessie and Houwelingen test [38] in the Design package (Version 2.3-0) in R Project Software (R Statistical computing 2012, Version 2.14.1 was used for all analyses).

**Table 1:** Description and hypothesized relationship for each of the variables considered in our statistical models.

Variable	Variable Code	Description	Hypothesis
Number of Water Sources	Num Source	Number of drinking water sources (0 = one source, 1 = multiple sources)	Dependent Variable
Treat Water	Treat Water	Whether a household filtered or boiled tap water before drinking (0 = No, 1 = Yes)	Dependent Variable
Institution Type	Water System	Which water system the household receives water from (i.e. PRASA = 0, Non-PRASA = 1)	+
Knowledge of Treatment	Treatment Knowledge	Identified if individual had knowledge of how institution (PRASA or Non-PRASA) treated water before it arrives at the tap (i.e. No = 0, Yes = 1)	-
Reported Problems with Institutional Management	Problems	Whether the respondent reported problems with the way institutions manage water (i.e. No = 0, Yes = 1)	+
Perceptions of Water Quality	Water Quality	Self-reported quality of drinking water from the tap (i.e. poor = 1, fair = 2, good = 3, excellent = 4)	-
Demographic Data	Age	Age	Control
Gender	Gender	Gender (0 = Male, 1 = Female)	Control

Variable, coding method, description, and the hypothesized relationship with the likelihood of adopting coping strategies for all covariates considered in both statistical models. A positive relationship indicates that the variable would lead to increased coping, as defined by a higher likelihood of treating water and obtaining water from multiple sources.

Finally, to assess the relative importance of each variable, we dropped each variable one at a time from the best logistic regression model and compared the AIC_c_ from the resulting model with the AIC_c_ from the best model. Variables that contributed most to model fit, and therefore were the most important in our analysis, had the largest change in AIC_c_ between the best model and the model with the variable in question dropped [39].

## Results

### ANOVA results

Several variables differed between PRASA and non-PRASA households (Table 2). Considering water management strategies, non-PRASA households were significantly more likely to obtain water from multiple sources, whereas PRASA households were significantly more likely to treat their tap water before drinking. This simple analysis suggests that households in PRASA and non-PRASA communities mitigate perceived low water quality in different ways. Considering perceptional variables, Non-PRASA households were significantly more likely to know how their institutions managed drinking water prior to distribution and non-PRASA households were also more likely to report higher water quality than PRASA households (Table 2).

**Table 2:** Comparison of each variable considered in our statistical models by institution type (PRASA vs non-PRASA).

	Mean value by Institution		ANOVA results		
Variable	PRASA	Non-PRASA	d, f	F	P
Number of Water Sources	0.06	0.26	1, 187	14.28	<0.001*
Treat Water	0.71	0.42	1, 187	16.26	<0.001*
Treatment Knowledge	0.49	0.73	1, 187	11.74	<0.001*
Problems	0.38	0.49	1, 187	2.47	0.12
Water Quality	2.41	2.98	1, 187	21.69	<0.001*
Age	53.20	50.25	1, 187	1.35	0.25

Mean value by institution (i.e. PRASA, Non-PRASA) and ANOVA results (degrees of freedom, F-statistic, p-value) are reported for each variable. * indicates p<0.05.

### Logistic regression models

**Figure 2:**
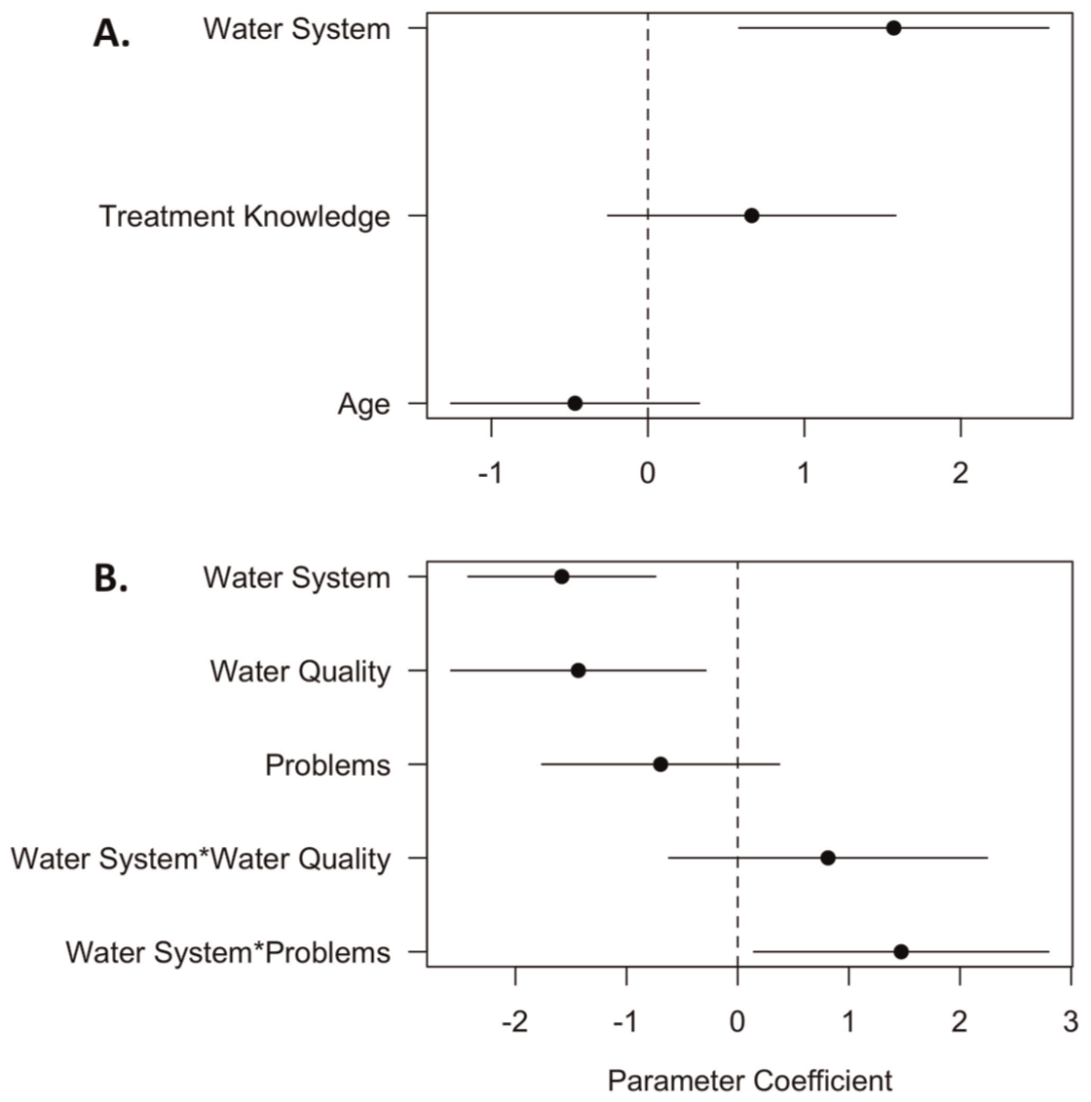
Parameter Estimate Plot of All Variables Considered in the Two Models that Predict Household Water Management Strategies. Standard errors are plotted as black lines. The variable is significant if standard error bars do not cross the zero axis. For the number of water sources (A), institution type is significant (p<0.005). For whether households treat water (B), institution type (p<0.001), perceptions of water quality (p<0.05), and the interaction between institution type and if households have a problem with institutional management (p<0.05) are significant.

The most important predictor of household decisions to obtain water from multiple sources was the institution that manages water (e.g. PRASA vs non-PRASA; Table 3, Figure 2A). Respondents in non-PRASA communities were more likely to obtain water from multiple sources than those from PRASA communities. Using the le Cessie and Houwelingen goodness of fit test, there is not a significant difference between observed and predicted values from the model suggesting good model fit (z = 0.78, sd = 0.19, p = 0.44).

**Table 3:** Results for each statistical model predicting which factors are associated with household water management strategies.

Response Variable	Covariates considered in logit model	Parameter Coefficient (Standard Error)	p value	N	GOF (p value)
Number of Water Sources	Water System	1.57 (0.52)	<0.005*	189	0.44
Number of Water Sources	Treatment Knowledge	0.66 (0.48)	0.17	189	0.44
Number of Water Sources	Age	−0.47 (0.42)	0.27	189	0.44
Treat Water	Water System	−1.58 (0.44)	<0.001*	189	0.19
Treat Water	Water Quality	−1.43 (0.60)	0.02*	189	0.19
Treat Water	Problems	−0.69 (0.56)	0.23	189	0.19
Treat Water	Water System*Water Quality	0.81 (0.75)	0.28	189	0.19
Treat Water	Water System*Problems	1.47 (0.70)	0.04*	189	0.19

Variables considered, parameter coefficients with standard error, p values, sample size, and goodness of fit for both of the full models including interaction terms. The first model predicts whether households obtain water from one or more sources, and the second model predicts whether households treat or do not treat their water. Significance of at least 5% is highlighted with a *.

The best predictors of household decisions to treat tap water before drinking were the institution that manages water, perceptions of water quality, and the interaction between the institution that manages water and problems with institutional management (Table 3, Figure 2B). PRASA households were significantly more likely to treat their water before drinking than non-PRASA households. Households that reported lower water quality were also more likely to treat their tap water, regardless of water management institution. Finally, the significant interaction between the institution that manages water and whether a household reported problems with institutional management suggests that non-PRASA households that had problems with institutional management were more likely to treat tap water before drinking than PRASA households. Le Cessie and Houwelingen goodness of fit test indicated a good fit between predicted and observed data (z = −1.31, sd = 0.14, p = 0.19).

### Variable importance

**Figure 3:**
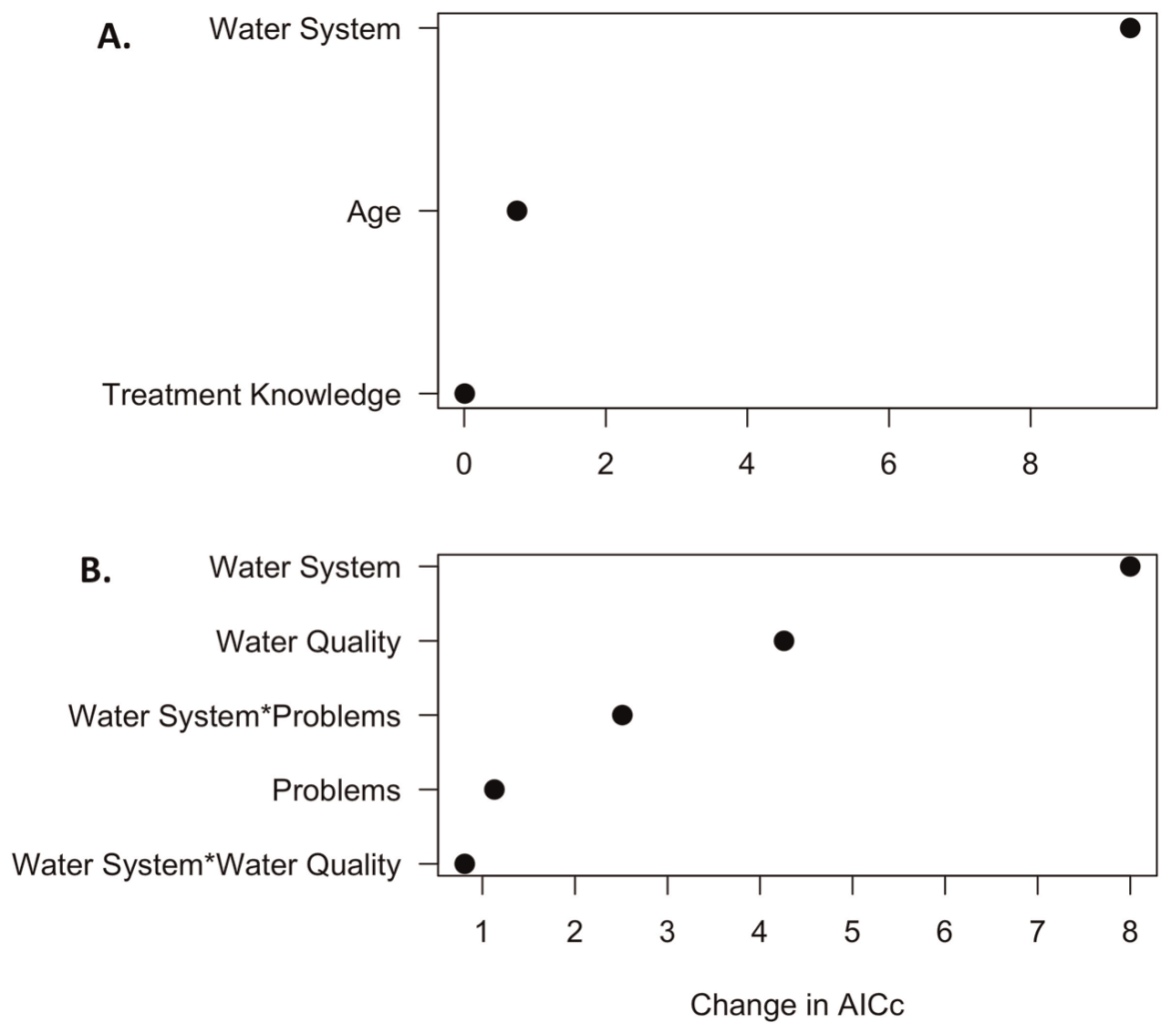
Importance of Each Covariate for Model Fit in the Two Models that Predict Household Water Management Strategies. Change in AIC_c_ for each of the covariates considered in the full logit model for the number of drinking water sources (A) and whether households treat or do not treat water (B). Larger changes in AIC_c_ values suggest that the variable contributed more to overall model fit. In both analyses (A and B), the institutional variable Water System (i.e. PRASA, non-PRASA) is the variable that contributes most to overall model fit. In the analysis of whether households treat water (B), water quality perceptions were also an important variable.

To understand the relative importance of each covariate considered in our logistic models (Table 1), we conducted a full model logistic regression and assessed the importance of each factor based on its contribution to model fit as measured by the change in AIC_c_ when that variable was dropped from the full model. In the model that predicted which households were more likely to obtain water from multiple sources, we found that the institution that manages water contributed most to model fit (Figure 3A). This suggests that whether households were from PRASA or non-PRASA communities was the most important variable for predicting whether households obtain water from multiple sources. The remainder of the variables in the model contributed little to model fit.

For the model that identified whether households treat or do not treat water, the institution that manages water was also the best predictor (Figure 3B). This suggests that whether households are from PRASA or non-PRASA communities was the most important variable to explain whether households treat or do not treat their water. Perceptions of water quality also contributed significantly to model fit (Figure 3B) suggesting that this variable is also important.

## Discussion

Policy-makers and agencies have increasingly recognized the importance of household water management for potable water provisioning given that households are the last point of management prior to consumption [13]. By understanding which factors most influence household water management, policy makers can better identify and target intervention strategies that improve access to clean drinking water. In this study, we examined household water management in rural Puerto Rico. It is important to understand household level management in these communities given that both government PRASA and community non-PRASA water treatment may be ineffective at providing clean water at the tap. Specifically, we analyzed (1) whether households obtained water from multiple sources or filtered or boiled tap water before drinking, and (2) which perceptional and socio-demographic factors were most associated with these management decisions. Our analysis suggests that three of our four initial predictions are correct: households manage water differently based on whether they are in PRASA or non-PRASA communities, households are more likely to treat water if they have problems with institutional management, and households are more likely to treat water if they believe that their tap water is of low quality (Figure 2). The fourth factor we predicted to be important in our analysis, whether households had knowledge of how water was treated prior to distribution, was not significant in our analyses.

The institution that manages water (i.e. PRASA vs non-PRASA) was the strongest driver of household drinking water management (Figure 3). PRASA households were more likely to filter or boil their water before drinking, whereas non-PRASA households were more likely to obtain water from multiple sources (Figure 2A). Differences in management strategies between PRASA and non-PRASA communities may be due to differences in perceptions of low water quality, possibly because of differences in water quality at the tap [14]. In PRASA communities, our informal discussions with community members indicate that perceptions of low water quality are due to the bad taste and odor of tap water, which community members attribute to over-chlorination. PRASA treatment facilities typically add chlorine to water prior to distribution, which has been associated with a reduction in bacteria such as *Escherichia Coli* (E. Coli) [40], [41]. However, based on our informal interviews with community members across our survey area, it is possible that PRASA systems are over-chlorinating water in this region; these anecdotal claims are bolstered by objective water quality measures collected by the government for the *barrios* (sub-districts) considered in our study, which show periods when chlorine levels are higher than those recommended by the EPA (> 4.0 ppm, Fig. S1) [24], [42], [43]. Thus, in PRASA communities, families may filter or boil their tap water in order to improve the smell and taste of water prior to consumption. In non-PRASA communities, discussions with community members suggest that perceptions of low water quality are due to turbidity, which community members attribute to the lack of treatment by non-PRASA institutions. Based on discussions with community members and the operators of non-PRASA systems, it appears as if water was not regularly treated (e.g. via chlorine addition or filters) in storage tanks prior to distribution, which resulted in increased water turbidity at the tap. Households mitigated this perceived low water quality by obtaining water from other sources, like store-bought bottled water or filtered water from friends and relatives in PRASA communities.

Second, households are more likely to treat water if they believe that their water was ineffectively managed by treatment facilities prior to distribution. This corroborates previous studies that show that households and communities increase water management efforts if they believe that government or private agencies ineffectively manage water prior to distribution [18], [19]. This result is only significant for non-PRASA communities (Figure 2B), suggesting that perceptions of institutional effectiveness drive decisions to treat water only in non-PRASA households. Institutional perceptions may play a stronger role in non-PRASA relative to PRASA communities because institutional management of drinking water is decentralized; given decentralized management, households in non-PRASA communities often play a stronger role in community-level water management than do households in PRASA communities, where water management is centralized within government agencies. Informal discussions with non-PRASA community members support this interpretation: non-PRASA households state that they feel a strong connection to water management institutions due to increased knowledge of treatment practices (Table 2) and the ability to participate in water management by speaking with local water operators or attending community meetings.

Finally, we found that perceptions of water quality were significant predictors of whether households were more likely to treat their water via filtering and boiling (Figure 2B). These results corroborate previous studies that find that households are more likely to manage their water if they perceive that their tap water is of low quality [15]. It is important to note that we only examined water quality perceptions and not objective water quality metrics at the household level, and it is unclear how well these two measures correlate with one another. If these two measures are not related, this could lead to water management decisions that result in low drinking water quality. For example, households may perceive that their water is of good quality, resulting in no treatment at the tap, when in reality objective water quality measures show that water treatment is required prior to consumption. Future studies should measure objective water quality standards in this region both before and after household treatment of drinking water to determine whether households are accurately perceiving low water quality and treating water effectively.

Based on the three main findings outlined above, we have several recommendations to improve water quality management in this region. First, we argue that both PRASA and non-PRASA institutions would likely improve water quality if they took household perceptions into account and understood how households manage water after it is distributed to the tap. Specifically, PRASA systems may improve water quality if they take steps to improve the taste and odor of tap water. If this low water quality is caused by over-chlorination as many people in PRASA communities believe, these systems should reduce the amount of chlorine used or let chlorinated water sit in storage tanks to allow for dechlorination prior to distribution while controlling for environmental variables that may increase chlorination byproducts [44]. Non-PRASA systems, on the other hand, may benefit by reducing the amount of turbidity at the tap, possibly by filtering water prior to distribution; this, and chlorination, may reduce perceived low water quality at the household scale. Second, objective water quality assessments should be coupled with these household level survey results to focus intervention strategies on the most vulnerable populations, particularly those households that have low water quality but do not treat their water or that treat their water ineffectively. For example, PRASA households perceive low water quality due to bad taste and odor possibly caused by over-chlorination, however, one of the main strategies to mitigate this problem is filtering tap water. Yet to dechlorinate water, expensive active carbon filters are required [45] and these filters were typically not used in this region, suggesting that household strategies to filter water may be ineffective at reducing chlorine content. Finally, given that perceptions of institutional effectiveness appear to influence household management decisions, particularly in non-PRASA communities, we argue that these agencies should strengthen perceptions of institutional effectiveness by increasing the involvement of local community members in water management decisions. If community members have an increased say in how water is managed prior to distribution, it is likely that there will be improved water management given that household-level concerns about water quality are more likely to be addressed [46], [47].

It is important to note that this study examined household perceptions of water quality and management, and it is possible that these perceptions are inaccurate when compared to objective measures. For example, most PRASA households believed that the bad taste and odor of tap water were caused by over-chlorination at treatment plants prior to distribution, but it is possible that the bad taste and odor were caused by other factors, like the addition of air or exposure to old pipes during the distribution process [48],[49]. Future work should quantify objective water quality and assess whether current management strategies are effective at improving water quality prior to consumption. Second, we conducted our analyses based on survey data collected for over 200 people who live in the Cayey Mountain range. It is possible that our results would differ if we increased the scope of this study, particularly to other regions in Puerto Rico that may have different management strategies in PRASA and non-PRASA systems. Future studies should conduct similar perceptional studies across the island to better identify how universal the findings of this study are. Finally, it is important to note that we used the broad category of non-PRASA to encompass a wide range of institutions. Given that non-PRASA management is decentralized and individual communities are making water management decisions, it is possible that each non-PRASA system managed water slightly differently prior to distribution. We argue, however, that the coarse institutional categorization of non-PRASA is important particularly for policy given that the government uses this coarse categorization in water quality and compliance monitoring [23]. Future work should examine the heterogeneity in water management across non-PRASA systems to identify whether certain management strategies result in different outcomes for water quality and management at the household scale.

In conclusion, this study highlights the importance of social surveys and decision-making analyses to better identify how households currently manage drinking water and which factors influence household management decisions. Our results suggest that both community-level properties, like the type of institution that manages water prior to distribution, and household-level factors, like water quality perceptions, are important for predicting household-level water management behavior. By understanding household perceptions of both water quality and treatment of water prior to distribution, policy-makers can better identify and target intervention strategies that are tailored to current household decision-making. This is important given that previous studies have suggested that policies have a higher chance of uptake and success if they are created considering the local context [20].

## Supporting Information

Figure S1 **Free chlorine levels in ppm in PRASA and non-PRASA communities across our survey area.** Data for PRASA communities were obtained from government databases collected at the barrio level, and data for non-PRASA communities were collected by our field team across several of our study communities of interest. These data suggest that free chlorine levels are typically lower in non-PRASA communities than PRASA communities, and several PRASA measurements have free chlorine levels higher than those recommended by the EPA (4.0 ppm, dotted horizontal line). This suggests that there may be over-chlorination in some PRASA communities.
(JPG)

Table S1 **Number of interviewees in Non-PRASA and PRASA communities in our two study municipalities.** We do not provide specific names of the communities or sectors surveyed in order to keep anonymity of our participants.
(JPEG)

## References

[1] Teunis P, Medema GJ, Kruidenier L, Havelaar AH (1997) Assessment of the risk of infection by Cryptosporidium or Giardia in drinking water from a surface water source. Water Research.

[2] Hellard ME, Sinclair MI, Forbes AB, Fairley CK (2001) A randomized, blinded, controlled trial investigating the gastrointestinal health effects of drinking water quality. Environ Health Perspect 109: 773–778. 11564611 10.1289/ehp.01109773 PMC1240403

[3] Prüss A, Kay D, Fewtrell L, Bartram J (2002) Estimating the burden of disease from water, sanitation, and hygiene at a global level. Environ Health Perspect 110: 537–542. 12003760 10.1289/ehp.110-1240845 PMC1240845

[4] Trevett AF, Carter RC, Tyrrel SF (2004) Water quality deterioration: A study of household drinking water quality in rural Honduras. International Journal of Environmental Health Research 14: 273–283. 15369992 10.1080/09603120410001725612

[5] Hunter PR, Toro GIR, Minnigh HA (2010) Impact on diarrhoeal illness of a community educational intervention to improve drinking water quality in rural communities in Puerto Rico. Bmc Public Health 10., 10.1186/1471-2458-10-219 PMC2876105 20426831

[6] Cash DW, Adger WN, Berkes F, Garden P, Lebel L, et al. (2006) Scale and cross-scale dynamics: Governance and information in a multilevel world. Ecology and Society 11..

[7] Berkes F (2006) From community-based resource management to complex systems: The scale issue and marine commons. Ecology and Society 11..

[8] Sarker A, Ross H, Shrestha KK (2008) A common-pool resource approach for water quality management: An Australian case study. Ecological Economics 68: 461–471.

[9] Lebel L, Garden P, Imamura M (2005) The politics of scale, position, and place in the governance of water resources in the Mekong region. Ecology and Society 10..

[10] Saravanan VS (2008) A systems approach to unravel complex water management institutions. Ecological Complexity 5: 202–215.

[11] Mintz E, Bartram J, Lochery P, Wegelin M (2001) Not Just a Drop in the Bucket: Expanding Access to Point-of-Use Water Treatment Systems. American Journal of Public Health 91: 1565–1570. 11574307 10.2105/ajph.91.10.1565 PMC1446826

[12] Clasen TF, Cairncross S (2004) Editorial: Household water management: refining the dominant paradigm. Tropical Medicine & International Health 9: 187–191. 15040554 10.1046/j.1365-3156.2003.01191.x

[13] Sobsey MD (2002) Managing water in the home: accelerated health gains from improved water supply. World Health Organization.

[14] Gartin M, Crona B, Wutich A, Westerhoff P (2010) Urban Ethnohydrology: Cultural Knowledge of Water Quality and Water Management in a Desert City. Ecology and Society 15: 36.

[15] Hu Z, Morton LW, Mahler RL (2011) Bottled Water: United States Consumers and Their Perceptions of Water Quality. Int J Env Res Pub He 8: 565–578. 10.3390/ijerph8020565 PMC3084479 21556204

[16] Fielding KS, Russell S, Spinks A, Mankad A (2012) Determinants of household water conservation: The role of demographic, infrastructure, behavior, and psychosocial variables. Water Resour Res 48..

[17] Sabau G, Haghiri M (2008) Household willingness-to-engage in water quality projects in western Newfoundland and Labrador: a demand-side management approach. Water and Environment Journal 22: 168–176.

[18] Zérah M (2000) Water, unreliable supply in Delhi. Manohar Publishers.

[19] Katuwal H, Bohara AK (2011) Coping with poor water supplies: empirical evidence from Kathmandu, Nepal. J Water Health 9: 143–158. 21301123 10.2166/wh.2010.151

[20] Jehu-Appiah C, Aryeetey G, Agyepong I, Spaan E, Baltussen R (2012) Household perceptions and their implications for enrolment in the National Health Insurance Scheme in Ghana. Health Policy and Planning 27: 222–233. 21504981 10.1093/heapol/czr032

[21] Molina-Rivera W (1998) Estimated water use in Puerto Rico, 1995. Washington D.C.: US. Geological Survey Open-File Report.

[22] Guerrero-Preston R, Norat J, Rodriguez M, Santiago L, Suarez E (2008) Determinants of compliance with drinking water standards in rural Puerto Rico between 1996 and 2000: a multilevel approach. P R Health Sci J 27: 229–235. 18782968 PMC13526437

[23] Quinones F (2005) PRASA has ample water supplies. Water Industry News.

[24] Environmental Protection Agency (2010) Puerto Rico Aqueduct and Sewer Authority (PRASA) Pollutant Discharge Settlement. Environmental Protection Agency.

[25] Toro GR, Minnigh HA (2004) Regulation and Financing of Potable Water Systems in Puerto Rico: A Study in Failure in Governance. AWRA Dunde: Scotland.

[26] de Cardenas SC (2011) Does private management lead to improvement of water services? Lessons learned form the experience of Bolivia and Puerto Rico. University of Iowa.

[27] Boose E, Serrano M, Foster D (2004) Landscape and regional impacts of hurricanes in Puerto Rico. Ecol Monogr 74: 335–352.

[28] US Bureau of the Census (2000) Census of population: social and economic characteristics. Washington D.C. USA: Department of Commerce, Economics, and Statistics Administration.

[29] Biernacki P, Waldorf D (1981) Snowball Sampling: Problems and Techniques of Chain Referral Sampling. Sociological Methods & Research 10: 141–163.

[30] North DC (1991) Institutions. The Journal of Economic Perspectives 5: 97–112.

[31] Makutsa P, Nzaku K, Ogutu P, Barasa P (2001) Challenges in implementing a point-of-use water quality intervention in rural Kenya. American Journal of Public Health 91: 1571–1573. 11574308 10.2105/ajph.91.10.1571 PMC1446827

[32] Peters RG, Covello VT, McCallum DB (1997) The determinants of trust and credibility in environmental risk communication: an empirical study. Risk Analysis 17: 43–54. 9131825 10.1111/j.1539-6924.1997.tb00842.x

[33] Palanski ME, Kahai SS, Yammarino FJ (2011) Team Virtues and Performance: An Examination of Transparency, Behavioral Integrity, and Trust. J Bus Ethics 99: 201–216.

[34] Jorgensen B, Graymore M, O'Toole K (2009) Household water use behavior: An integrated model. Journal of Environmental Management 91: 227–236. 19788952 10.1016/j.jenvman.2009.08.009

[35] Below TB, Mutabazi KD, Kirschke D, Franke C, Sieber S, et al (2012) Can farmers' adaptation to climate change be explained by socio-economic household-level variables? Global Environmental Change 22: 223–235.

[36] Hurvich C, Tsai C (1989) Regression and Time-Series Model Selection in Small Samples. Biometrika 76: 297–307.

[37] Gelman A, Hill J (2007) Data Analysis Using Regression And Multilevel/Hierarchical Models. Cambridge Univ Pr.

[38] le Cessie S, van Houwelingen J (1991) A Goodness-of-Fit Test for Binary Regression-Models, Based on Smoothing Methods. Biometrics 47: 1267–1282.

[39] Burnham KP, Anderson DR (2002) Model Selection and Multimodel Inference. 2nd ed. New York: Springer.

[40] Payment P, Trudel M, Plante R (1985) Elimination of viruses and indicator bacteria at each step of treatment during preparation of drinking water at seven water treatment plants. Appl Environ Microbiol 49: 1418–1428. 2990337 10.1128/aem.49.6.1418-1428.1985 PMC241740

[41] Arnold BF, Colford JM (2007) Treating water with chlorine at point-of-use to improve water quality and reduce child diarrhea in developing countries: a systematic review and meta-analysis. Am J Trop Med Hyg 76: 354–364. 17297049

[42] Autoridad de Acueductos y Alcantarillados (2001-2010) Water Quality Reports.

[43] Puerto Rico Department of Public Health (1996-2009) Water Quality Reports.

[44] Chowdhury S, Champagne P, McLellan PJ (2009) Science of the Total Environment. Science of the Total Environment, The 407: 4189–4206. 10.1016/j.scitotenv.2009.04.006 19419751

[45] Worley JL (2000) Evaluation of Dechlorinating Agents and Disposable Containers for Odor Testing of Drinking Water. Virginia Polytechnic Institute and State University.

[46] Olsson P, Folke C, Berkes F (2004) Adaptive Comanagement for Building Resilience in Social-Ecological Systems. Environmental Management 34: 75–90. 15383875 10.1007/s00267-003-0101-7

[47] Larson AM, Soto F (2008) Decentralization of Natural Resource Governance Regimes. Annu Rev Env Resour 33: 213–239.

[48] Sangodoyin AY (1993) Water quality in pipe distribution systems. Environmental Management and Health.

[49] Young WF, Horth H, Crane R, Odgen T, Arnott M (1996) Taste and Odour Threshold Concetrations of Potential Potable Water Contaminants. Water Research 30: 331–340.

