# Supplementary figures and images for: Perceptional and Socio-Demographic Factors Associated with Household Drinking Water Management Strategies in Rural Puerto Rico
Source: PLoS One. 2014 Feb 28;9(2):e88059. doi: 10.1371/journal.pone.0088059 (PMC3938413; doi:10.1371/journal.pone.0088059)

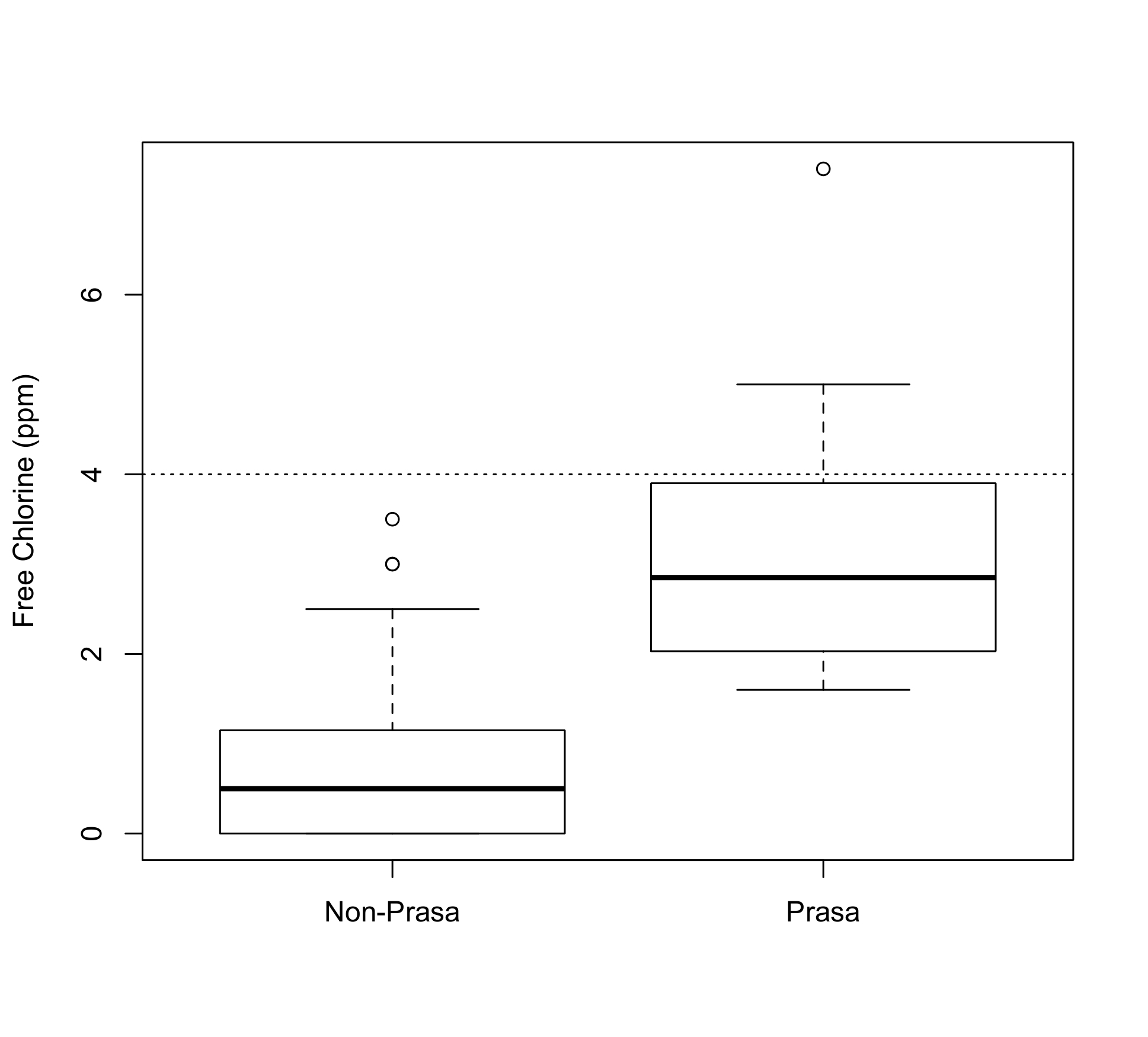

Supplement: Figure S1 — Free chlorine levels in ppm in PRASA and non-PRASA communities across our survey area. Data for PRASA communities were obtained from government databases collected at the barrio level, and data for non-PRASA communities were collected by our field team across several of our study communities of interest. These data suggest that free chlorine levels are typically lower in non-PRASA communities than PRASA communities, and several PRASA measurements have free chlorine levels higher than those recommended by the EPA (4.0 ppm, dotted horizontal line). This suggests that there may be over-chlorination in some PRASA communities. (JPG) [file pone.0088059.s001.jpg]

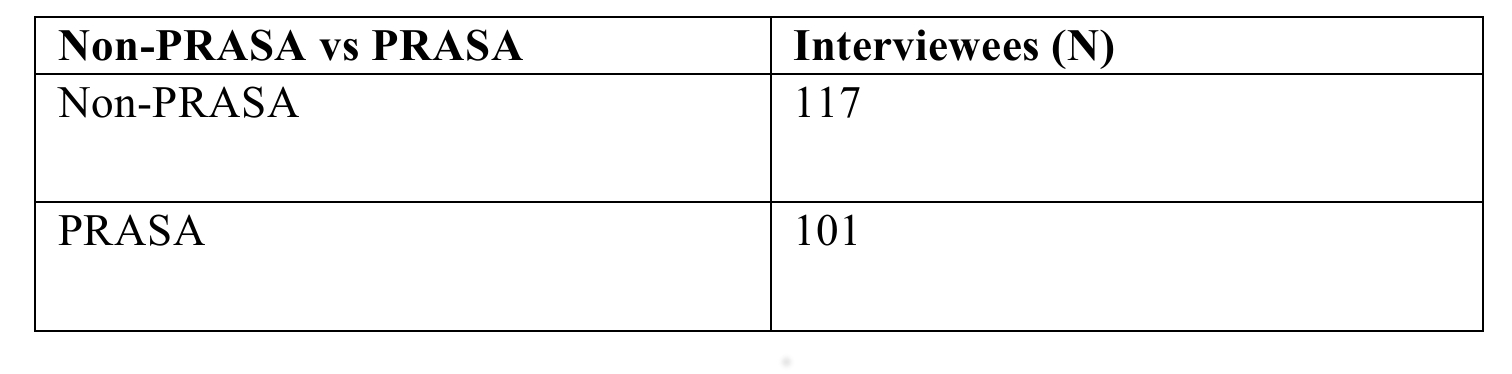

Supplement: Table S1 — Number of interviewees in Non-PRASA and PRASA communities in our two study municipalities. We do not provide specific names of the communities or sectors surveyed in order to keep anonymity of our participants. (JPEG) [file pone.0088059.s002.jpeg]
